# Multiple Resource Use Efficiency (*m*RUE): A New Concept for Ecosystem Production

**DOI:** 10.1038/srep37453

**Published:** 2016-11-21

**Authors:** Juanjuan Han, Jiquan Chen, Yuan Miao, Shiqiang Wan

**Affiliations:** 1 International Center for Ecology, Meteorology, and Environment, Nanjing University of Information Science and Technology, Nanjing 210044, China; 2 CGCEO/Geography, Michigan State University, East Lansing, Michigan 48824, USA; 3 International Joint Research Laboratory for Global Change Ecology, State Key Laboratory of Cotton Biology, College of Life Sciences, Henan University, Kaifeng, Henan 475004, China

## Abstract

The resource-driven concept, which is an important school for investigating ecosystem production, has been applied for decades. However, the regulatory mechanisms of production by multiple resources remain unclear. We formulated a new algorithm model that integrates multiple resource uses to study ecosystem production and tested its applications on a water-availability gradient in semi-arid grassland. The result of our experiment showed that changes in water availability significantly affected the resources of light and nitrogen, and altered the relationships among multiple resource absorption rate (ε), multiple resource use efficiency (*m*RUE), and available resource (R_avail_). The increased water availability suppressed ecosystem *m*RUE (i.e., “declining marginal returns”); The changes in *m*RUE had a negative effect on ε (i.e., “inverse feedback”). These two processes jointly regulated that the stimulated single resource availability would promote ecosystem production rather than suppress it, even when *m*RUE was reduced. This study illustrated the use of the *m*RUE model in exploring the coherent relationships among the key parameters on regulating the ecosystem production for future modeling, and evaluated the sensitivity of this conceptual model under different dataset properties. However, this model needs extensive validation by the ecological community before it can extrapolate this method to other ecosystems in the future.

Biophysical regulations and resource-driven investigations are the two primary approaches for modeling the magnitudes and dynamics of ecosystem production and understanding its mechanisms^1^. In general, ecosystem production is influenced by the complex interactions of biology, environment and disturbances^2^ (i.e., biophysical regulation). If based on economic principles, ecosystem production can be also interpreted as an output of carbohydrates and other organic compounds synthesized from the input source of water, CO_2_, and nutrients^3^ (i.e., resource-driven concept). To continue the analogy, various available resources are treated as “investments”, gross primary production (GPP) as “revenue” and the net primary production (NEP) as “profit”. In practice, one perused a maximized profit would tend to get more production under the limited multiple resources^4^. However, in recent decades, ecosystem studies have been predominantly conducted with empirical, theoretical, or modeling endeavors based on biophysical regulations from various processes, whereas resource-driven investigations lag significantly behind.

Extensive research has demonstrated that resource use efficiency varies by resource type (e.g., nutrient, light, and water), ecosystem type, physical condition (e.g., soil and climate) and location, with uneven consequences on ecosystem production^5^,^6^,^7^. In general, a highly available resource associated with more production is expected to possess either a high or near constant resource use efficiency. A previous study across biomes indeed verified that high rainfall (i.e., high water availability) contributes to greater dry matter given a certain amount of absorbed resources^5^. For example, forests generally have higher water-use efficiency (WUE) than grasslands^8^. This phenomenon has also been reported in many ecosystem-scale studies, with most focusing on available light and nitrogen in forests. Light-use efficiency (LUE) is highly correlated with photosynthetically active radiation (PAR)^9^. Plants in fertile soils show stronger responses to nutrient addition (i.e., nutrient availability) than those in infertile soils^10^, resulting in a higher nitrogen-use efficiency (NUE)^11^ and consequently promoting ecosystem production^12^,^13^. However, other studies have reported an inverse response of resource use efficiency (RUE) to available resources—a phenomenon known as “declining marginal returns” that describes that the increased rate of ecosystem production (i.e., RUE), due to the addition of a single resource, will progressively decline compared to the previous rate. For example, elevated precipitation (i.e., water availability) can substantially lower the WUE in grasslands^14^ and wheat^15^, and the enhanced nitrogen supply can significantly reduce NUE in a forest plantation^16^. In tropical montane forests, instantaneous phosphorus-use efficiency (PUE) decreases with the gradual increase in phosphorus^17^. Clearly, the ecosystem production is often regulated by multiple resources, and the single resource use process was generally constrainted by the other resources. Therefore, the availability and efficiency of an individual resource may not reflect the overall RUE for any ecosystem.

Multiple resources usually change synchronously^4^; the changes in one resource will likely induce changes in other resource uses at leaf, species, and community levels and at different temporal scales. Using the experimental data from *Acacia auriculiformis*, Phillips and Riha^18^ demonstrated that drought stress can reduce absorbed photosynthetically-active radiation (aPAR) and LUE, but can enhance WUE, implying that, at the annual scale, the shift in one resource availability would cause disproportionate consequences on other resource use efficiency. Doubled CO_2_ concentration can increase annual LUE and NUE with different magnitudes, the LUE being higher in the later than early stage of canopy, while NUE being greater in early than later stage at community scale^19^. At shoot scale, great transpiration tends to promote NUE, but reduce WUE and LUE in a mixed coniferous stand^20^. Similarly, elevated temperature can increase LUE by 8–9% but reduce WUE by 19–34% in a pure Scots pine (*Pinus sylvestris*) stand^21^. These conditional relationships among multiple resources also exist at species level. Field *et al.*^22^ reported an inverse relationship between photosynthesis NUE and photosynthesis WUE in evergreen vegetation; and Reich *et al.*^23^ found significantly negative relationships between photosynthesis NUE and WUE in an elm stand. Nevertheless, these studies fail to systematically explore the influence paths or mechanisms about how one resource, use efficiency, affect the others by including intermediate variables (e.g., absorbed light, soil water or nitrogen), when the ecosystem was directly triggered by one resource change.

The economic principle in ecosystem production emphasizes a higher production return with a relatively low investment. A greater multiple RUE (*m*RUE) does not always warrant high production. For example, declined water availability in a semi-arid grassland generally promote WUE, but ecosystem production does not increase as expected^14^. As the result, we infer that the magnitudes and dynamics of production probably depend on the independent and interactive effects of multiple resource absorption rate (ε) and *m*RUE. From the viewpoint of plant physiology, an increase in water availability in soil would probably enhance the water uptake and soluble materials (e.g., nitrogen) of the plant through transpiration pull, leading to a higher ε. Meanwhile, the *m*RUE may be reduced if the amount of absorbed resources surpasses the demand of plants^24^, suggesting that the increased ε combined with the decreased *m*RUE may still enhance plant production. Previous studies have documented that the increased ε of water^25^ and nitrogen^26^ would stimulate *m*RUE, suggesting that ε and *m*RUE may not independently regulate ecosystem production. Unfortunately, few studies have examined the complex causal relationships between ε and *m*RUE in mediating ecosystem production. Distinguishing the independent and interactive effects of ε and *m*RUE in regulating ecosystem production will help to fill these knowledge gaps.

The objective of this study was to establish a new conceptual model by integrating multiple resources on ecosystem production, quantify the dynamics of available resources, ε, and *m*RUE, and promote the potential applications of *m*RUE in ecosystem ecology. In this scientific adventure for understanding the relations between *m*RUE and ecosystem production, the field data from a water-availability gradient with increased and decreased precipitation in a semi-arid grassland on the Mongolia Plateau were used to illustrate the model applications. The specific questions of this study were: (i) how does water availability affect other resources and contribute to the variations of ε and *m*RUE; and (ii) is there a causal relation between ε and *m*RUE in regulating the production?

## Developing the multiple-resource use efficiency (*m*RUE) model

Environmental resources have long been recognized as one of the important proxies regulating ecosystem production. The LUE model utilizes the principle of fixed carbohydrate as output energy from solar radiation in estimating gross primary production^27^:


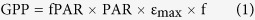


where GPP is gross primary production, fPAR is the fraction of absorbed PAR (MJ m^−2^) by vegetation, PAR is incident photosynthetically active radiation (MJ m^−2^) during a certain time period, ε_max_ is potential LUE (gC m^−2^ MJ^−1^) that can be scaled to a range of 0–1 using plant caloric values, and f is a scalar limiting ε_max_ from other environmental drivers.

Based on the Liebig Law, which states that ecosystem production is regulated by limited cues, light resource is not expected to be the sole driving force in some ecosystem types. Binkley *et al.*^25^ synthesized published results from a *Eucalyptus* plantation, extending the concept of RUE to include water, nutrients and light use as:


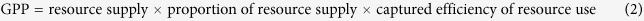


These two schools of thought, although expressed differently, have many similarities and can be united under RUE_*i*_ when individual resource is considered. RUE_*i*_ as the accumulated dry matter per unit of absorbed multiple resources. Production is often expressed as the dry matter, such as GPP, net primary production (NPP), or aboveground net primary production (ANPP). Using annual ANPP as an example, RUE*i* can be modeled as:


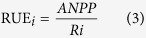


where R_*i*_ is the amount of absorbed resources within a year. RUE_*i*_ can be applied to specific terms, such as water (WUE = ANPP:Tr, where Tr is the transpiration), nitrogen (NUE = ANPP:N_uptake_, where N_uptake_ is the absorbed nitrogen amount), and light (LUE = ANPP:aPAR, where aPAR is the absorbed PAR).

The absorbed resources (R_*i*_) can be further expressed as two quantitative variables, R_avail_ and ε_*i*_ as follow:


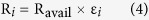


Combining Eq. (4) with Eq. (3), we have:


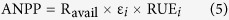


where ANPP is regulated by an individual resource, such as light, water and nitrogen:


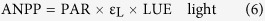



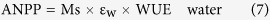



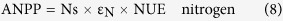


where PAR, Ms and Ns are accumulated available resources at the annual scale. Ms is the accumulated soil water content. Ns is the accumulated available nitrogen in soil (e.g., NO_3_^−^, NH_4_^+^), and this variable was substituted by soil total nitrogen content in this study. ε_L_, ε_w_, and ε_N_ are ratios of absorbed light, water, and nitrogen, respectively. RUE_*i*_ and ε_*i*_ are the ratios with a range of 0–1. The units of PAR and aPAR can be converted to g m^−2^, if divided by plant caloric values. The units of other variables, except RUE_*i*_ and ε_*i*_, are also in g m^−2^. In this study, we used 19.38 kJ g^−1^ as the caloric value in a semi-arid grassland^28^ (see the supplemental materials for details).

**Figure 1:**
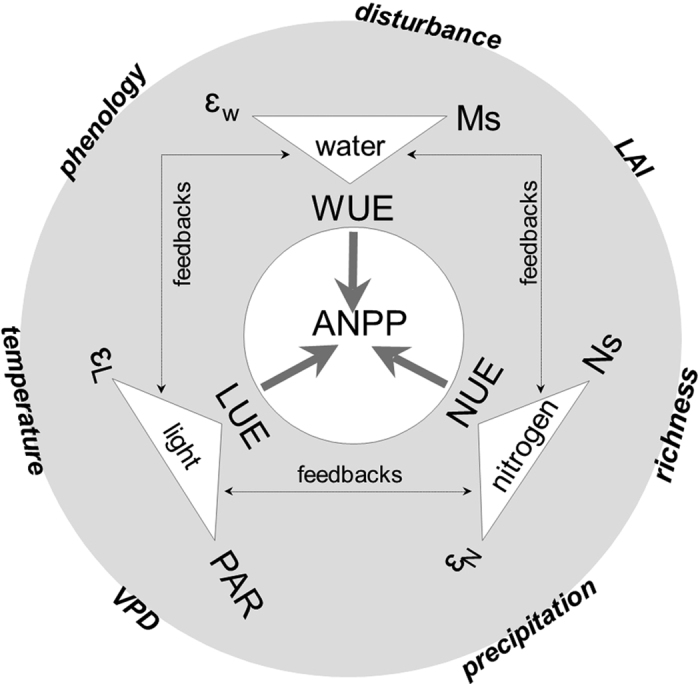
The conceptual framework of this study. Within the matrix of the bio-physical environment (microclimate and disturbance), the magnitude of aboveground net primary productivity (ANPP) is determined by the resource use matrix of [ε, *m*RUE, R_avail_] and their complex interactions. For each type of resource, there exists complex interactions among [ε, *m*RUE, R_avail_] at various temporal scales. Alteration of any element of the resource use matrix will trigger changes in other elements. This study will examine the feedbacks among the elements, with a focus on water, light, and nitrogen.

When more resources are involved, ANPP is regulated as:


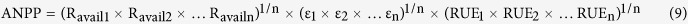


where ε_*i*_ = Tr:Ms = N_uptake_:Ns = aPAR:PAR. In Eq. (9), the integrated term of multiple RUE_*i*_ (i.e., (RUE_1_ × RUE_2_ × …RUE_n_)^1/n^) refers to *m*RUE, and that of multiple ε_i_ (i.e., (ε_1_ × ε_2_ × …ε_n_)^1/n^) refers to ε. Compared with Binkley’s model, our multiple resource model (Eq. (9)) included the interactions among the drivers and constraints of one resource on another. Another advantage of this model is that these resources may interactively determine ANPP, although one or some resources may dominate the magnitudes and directions of others as these resources are limiting factors (Fig. 1). We used the data collected in a field experiment with precipitation manipulation in semi-arid temperate grassland to test the potential use of this new *m*RUE model (Eq. 9). The major acronyms were shown in Table 1.

## Coherent relationships among the key parameters

To examine the complex interactions among the elements of multiple resources (i.e., ε, *m*RUE, R_avail_), a non-recursive structural equation model (SEM) for our experimental data in the semi-arid grassland (Fig. 4a without coefficients) was constructed to test four specific hypotheses:

1. In a water-limited environment, increased available water significantly stimulates absorbed light (Ms → aPAR)^29^. Associated with the process of water uptake by roots, the mass flow of mineral nutrient simultaneously transplant to different organs in plants (Tr → N_uptake_). The absorbed nutrient usually acts as catalyst (at the scale of leaves and seconds) and maintains nutrient demand for plant growth (at individuals and community levels), leading to more photosynthetic products, which will indirectly increase the absorbed PAR (N_uptake_ → aPAR)^12^.
2. ε is a function of absorbed resources and available resources and, thus, is significantly affected by available soil water (Ms → ε) and by absorbed water, light, and nitrogen (Tr → ε; aPAR → ε; N_uptake_ → ε)^25^. At the ecosystem level, ε is expected to have significant effects on *m*RUE (ε → *m*RUE).[Table t1]
3. The economic principles of “declining marginal returns”, which describes the decline of productivity or dry matter per unit of absorbed (*m*RUE) when the supply of single resource increases, can be applied for explaining *m*RUE (Ms → *m*RUE; aPAR → *m*RUE; N_uptake_ → *m*RUE)^25^,^30^,^31^.
4. There may exist a feedback between RUE and ε, which is responsible for the changes in production (*m*RUE → ε).

## Resource use efficiency among ecosystems

Our case study in the semi-arid grassland of the Mongolia Plateau showed that there were lower use efficiencies of water, light, and nitrogen than those found in most ecosystems, with the mean WUE, LUE and NUE values in the control plots of 0.05%, 1.2%, and 62.47, respectively (Table 2). The WUE in this system was significantly lower than that in a forest at the annual scale^32^ and in some grasslands at daily scales^33^, but it was close to the maximum WUE of 0.042% in the driest years for arid ecosystems^34^. In other words, the annual mean WUE of this arid grassland was close to the potential maximum WUE of arid biomes, further supporting the consensus on water sensitivity (i.e., WUE) in these water-limited ecosystems. However, light did not appear to be limiting production in this semiarid grassland. In addition, there appeared a lower LUE than that in forests^5^,^29^, croplands^3^, and in other grasslands^35^. The NUE in our system was substantially higher than that in crops, likely because the overcropped and unsuitable management practices in crops might have reduced available nutrients and aggravated leaching processes^36^. The NUE in this grassland was also lower than those in a forest^31^ and in a semi-arid grassland on the Mongolia Plateau^37^.

## Complex interactions among the elements of *m*RUE

**Figure 2:**
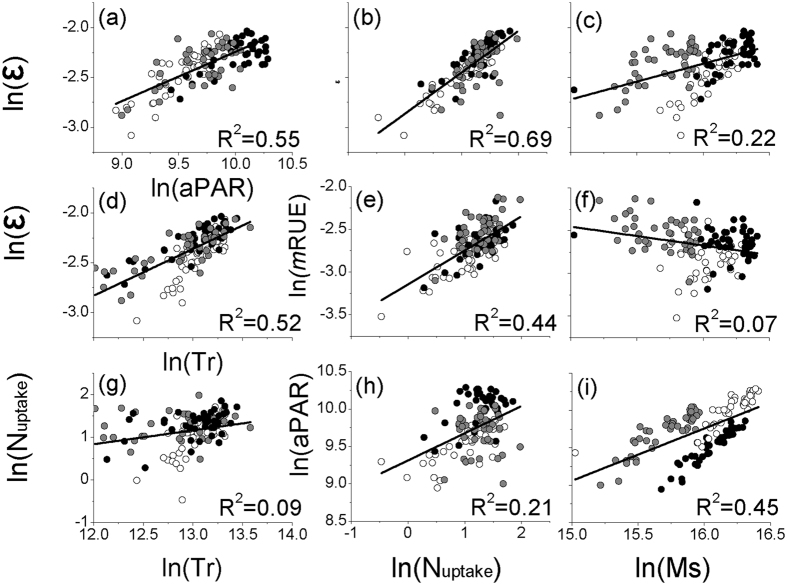
The bivariate correlations in the SEM model when the data were log-transformed. The colored dots indicated variables measured in different years, where grey, black, and white mean 2010, 2011, and 2012, respectively. The units of aPAR, Ms, Tr, N_uptake_ were g m^−2^ year^−1^.

Water availability was responsible for the large changes in resource use and efficiency in the semi-arid grassland, including ε, *m*RUE, and R_avail_ in this study. Experimental manipulation with both increased and decreased precipitation constituted for a wide spectrum of water availability, ranging from 70 mm to 345 mm prior to measuring ANPP, producing significant changes in water, light, and nitrogen resources. Among these available or absorbed resources, Pearson correlations revealed that N_uptake_ had a linear relationship with Tr (Fig. 2g, R^2^ = 0.09). aPAR showed significantly positive correlations with N_uptake_ (Fig. 2h, R^2^ = 0.21) and Ms (Fig. 2i, R^2^ = 0.45), with a stronger effect on Ms than N_uptake_, suggesting a greater response of aPAR to water than nitrogen resource. These correlations indicated positive relationships among these three resources. However, this conclusion was inconsistent with some previous studies, where one resource was reported to have opposite correlations with other resources^22^. As for ε and *m*RUE, ε was linearly correlated with the absorbed light (aPAR; Fig. 2a, R^2^ = 0.55, Fig. 4b, spurious R^2^ = 0.16), nitrogen (N_uptake_; Fig. 2b, R^2^ = 0.69, Fig. 4b, spurious R^2^ = 0.32) and water (Ms: Fig. 2c, R^2^ = 0.22, Fig. 4b, spurious R^2^ = 0.04; Tr: Fig. 2d, R^2^ = 0.52, Fig. 4b, spurious R^2^ = 0.10) by plants. *m*RUE also showed linear dependence upon the absorbed nitrogen (N_uptake_; Fig. 2e, R^2^ = 0.44, Fig. 4b, spurious R^2^ = 0.038) and water (Ms; Fig. 2f, R^2^ = 0.07).

**Figure 3:**
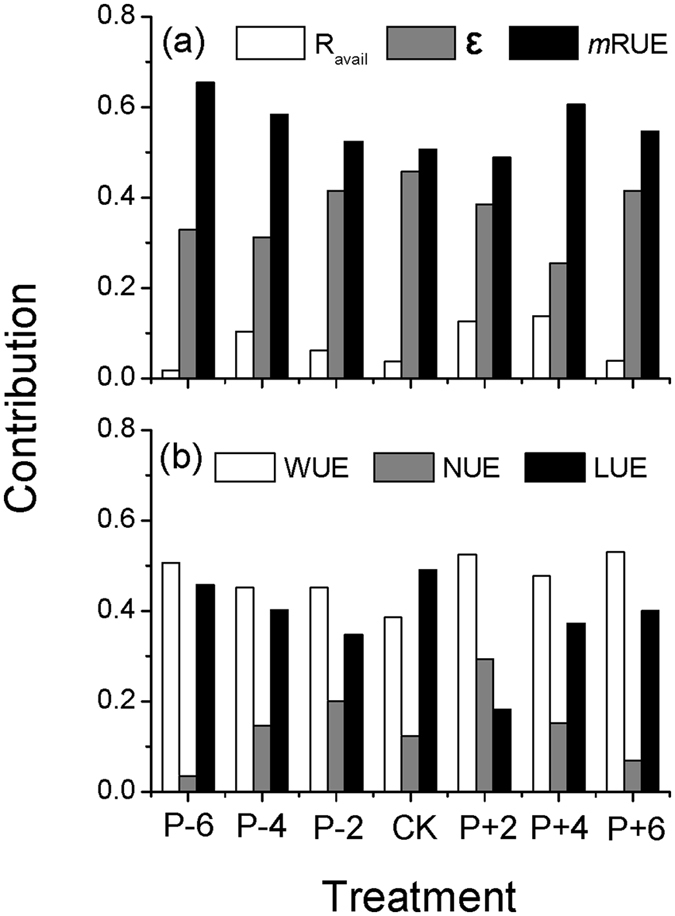
The relative contributions to ANPP and *m*RUE from the model elements.

When distinguishing the relative contributions of ε and *m*RUE to ANPP, we found that ε and *m*RUE had dominant roles in regulating ANPP under all the treatments, with greater contribution from *m*RUE than that from ε (Fig. 3a). WUE, LUE, and NUE accounted for, on average, 47.55%, 37.90%, and 14.55% of the variations of *m*RUE across the treatments, respectively (Fig. 3b). These results suggest that the resource absorption ratio and efficiency were critical factors in determining ecosystem production, and that the driving factor (i.e., water resource) always produce a main effect on multiple RUE.

## Tradeoff effects of ε and *m*RUE on production

**Figure 4:**
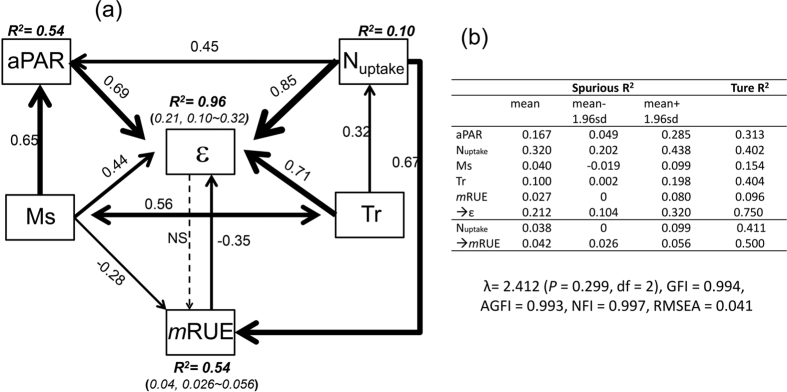
The left panel was the non-recursive SEM model. All the variables are observed indicators (in boxes). Dash line referred to insignificant correlation between these two variables, “NS” meant insignificance. A set of model performance indexes, such as λ = 2.412 (*P* = 0.299, df = 2), GFI = 0.994, AGFI = 0.993, NFI = 0.997, RMSEA = 0.041, and stability index = 0.025, suggested that this model was fitted well with our data. The determination coefficient (R^2^) represented the overall explanation on this dependent variable, and the other coefficients were correlation coefficients (r). We should note that these coefficients were calculated based on the experimental data (after log-transformed), so the spurious correlations were unavoidable. Thus, the right panel pointed out these spurious determination coefficients when the X and Y variables are non-independent.

The water-driven changes in other two resources (i.e., light and nitrogen) significantly affected ε (Fig. 4, Table 3). Plant transpiration directly promoted ε (β = 0.43, r = 0.71, spurious R^2^ = 0.1 × 28% = 0.028) but was greatly offset by the direct effect of available water (β = −0.15, r = 0.44, spurious R^2^ = 0.04 × 5% = 0.002), contributing to a total effect of 0.28 (Table 3: β = 0.43 + (−0.15) = 0.28, spurious R^2^ = 0.03). In addition to the direct effects, water resource indirectly stimulated ε via regulating absorbed light and nitrogen (Table 3: β = 0.20 + 0.25 = 0.42, spurious R^2^ = 0.062). Therefore, there was a greater contribution from water-induced changes in light and nitrogen than that from the water resource directly. In addition, water, light and nitrogen resources collectively explained 75% (96–19% (spurious R^2^) = 77%, Fig. 4b) of the variations of ε. Our results are consistent with previous studies that water addition may significantly enhance leaf area index of forest canopy^13^ and aPAR^29^. However, these studies emphasize the ε responses to individual resources only, which would lead to inconsistent results from multiple resources. In conclusion, understanding the influence paths and processes of the multi-resource absorption rate would be necessary to comprehensively characterize and explore the change dynamics and control mechanisms over ε.

We confirmed our third hypothesis regarding the application of “declining marginal returns” in modeling ecosystem production from multiple resource use efficiency (Fig. 4). This concept was initially proposed for modeling the instantaneous photosynthesis process at leaf level by Ögren and Evans^38^, who hypothesized that the increase in the photosynthesis rate would decrease with incident light exceeding the demand (i.e., decreases on instantaneous energy-use efficiency, i.e. LUE). In our study, we found that available water directly reduced *m*RUE (Table 3: β = −0.49, Fig. 4a: r = −0.28) and transpiration indirectly promoted *m*RUE via increasing plant nitrogen content (i.e., N_uptake_, β = 0.32×0.66 = 0.21, spurious R^2^ = 0.013). The total effects of water resource eventually reduced *m*RUE by 28% (Table 3: β = −0.49 + 0.21 = −0.28), jointly contributing to about 50% of the total variations when we excluded the spurious contribution (mean spurious R^2^ = 0.042, Fig. 4b). Our results demonstrated a response from *m*RUE to an individual resource that was similar to the responses of individual RUEs found in literature. Nevertheless, our conclusion may not be extrapolated to all ecosystem types, particularly for forests. For example, Binkley *et al.*^39^ reported that a more productive site had higher absorption rate and resource use efficiency in an *Eucalyptus* plantation. Also, Balster and Marshall^12^ reported that fertilization promoted the ratio of converted stemwood in Douglas-fir stands (*Pseudotsuga mensizii*). Thus, our *m*RUE model—an alternative for characterizing the relationships between resource use efficiency and available resources—would be ecosystem specific, because of ecosystem-specific regulations and the complex interactions with other biophysical drivers.

**Figure 5:**
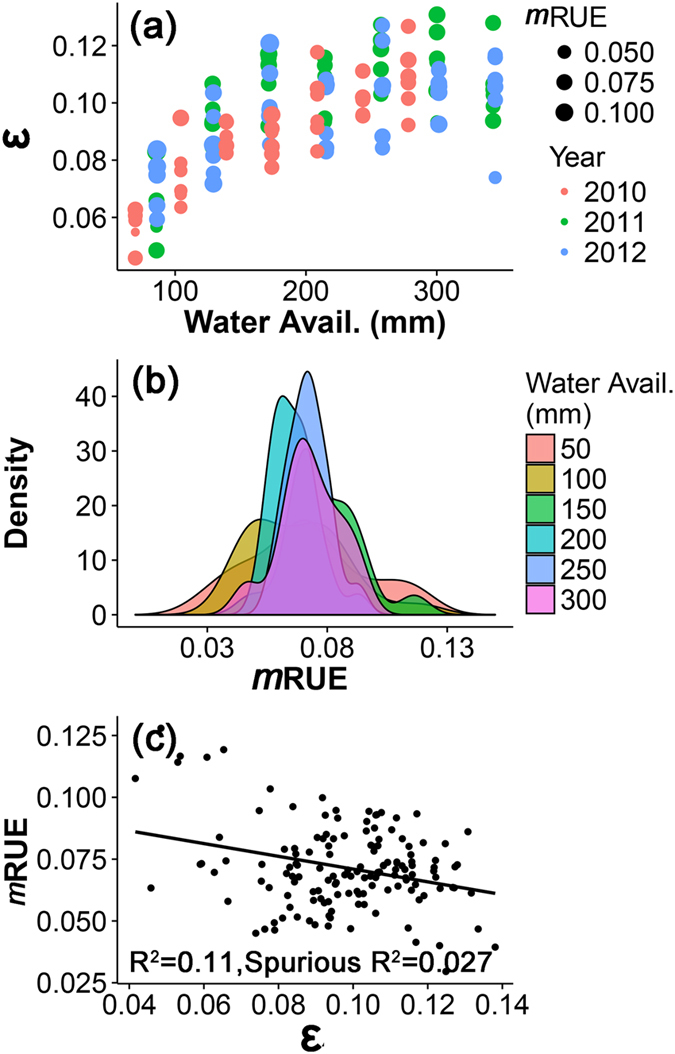
The dynamic variations of ε and *m*RUE (**a**,**b**) along the water availability gradient (Water Avail.) and their correlation (**c**).

The ε and *m*RUE presented different distributions along the water availability gradient. The ε increased significantly along the water availability gradient; but over three years, it showed distinct levels (Fig. 5a). *m*RUE did not show a similar trend with ε, but had different probability distributions (i.e., density) created by different ranges of water-availability (Fig. 5b). Moreover, *m*RUE had a negative correlation with ε (Fig. 5c).

This negative correlation did not imply that water-induced changes in ε had significant influences on *m*RUE; but instead, *m*RUE indeed produced a negative effect on ε (*i.e.,* one-way causality) (Fig. 4a). This phenomenon is in agreement with the classical economic principle of “inverse feedback”^40^. In our study, the semi-arid ecosystem encountered intense stress due to water availability, the *m*RUE, as one of the regulatory factors of ecosystem production, would represent a negative feedback to other biological cues (e.g., ε) to alleviate its fluctuation on ecosystem production. Specifically, water resource had a significantly positive influence on ε (Table 3: β = (−0.15) + 0.20 + 0.43 + 0.25 = 0.73, spurious R^2^ = 0.092) and a negative effect on *m*RUE (Table 3: β = (−0.49) + 0.21 = −0.28, spurious R^2^ = 0.013), suggesting that the increase in water resource (i.e., Ms and Tr) can reduce *m*RUE dramatically, but enhance ε. In addition, ε was further elevated by the negative effects from *m*RUE (*m*RUE → ε, β = −0.11) to ease the negative changes of *m*RUE on production and ultimately promoted the production under the increased water availability. These tradeoff effects between ε and *m*RUE determined that the increased single resource availability would promote ecosystem production rather than decrease it, even when the *m*RUE reduced. Thus, the processes of “declining marginal returns” and “inverse feedback” jointly explain this phenomenon in nature. For example, forests are always more productive than grasslands, and fertile soil is always more productive than infertile soil for a given ecosystem.

## Sensitivity analysis on *m*RUE model

**Figure 6:**
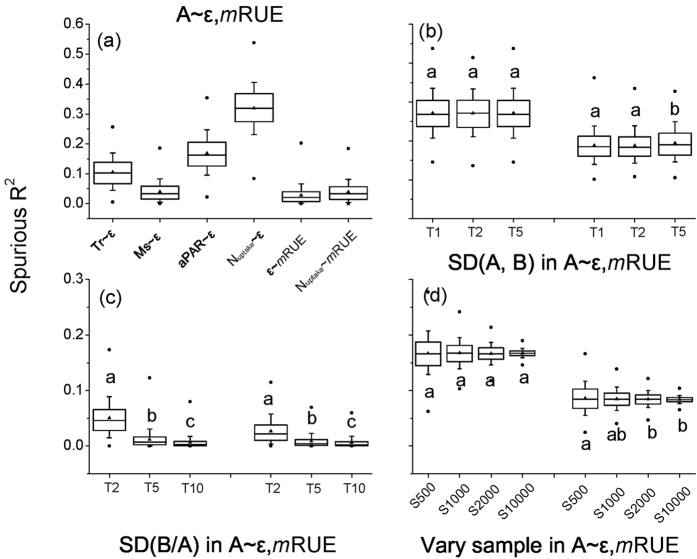
The spurious determination coefficient (R^2^) in the experiment (**a**) and several possible situations of SEM model (**b**–**d**) using stochastic simulations. All simulations ran 1000 times. Box-and-whisker plots represented the median (horizontal bar), mean (triangle), the 25^th^ and 75^th^ percentiles (box range), 10^th^ and 90^th^ percentiles (whisker), and the maximum and minimum outliers (point) of their distributions. The different lower case letters meant significant differences, and the same one or two letters meant insignificant difference between them. A variable was set as the common variable in ε or *m*RUE, B was set as the independent variable for calculating ε or *m*RUE. In subplot (**a**) we used normal random numbers, which have an identical mean, standard deviations (SD) and sample size (n = 126) as our experiment. In subplots (**b**,**c**), we used the normal random number with the sample size of 200 and mean of 20. Additionally, we set the initial SD of A as 1. In subplot (**b**), the varied ranges of spurious R^2^ were produced when the standard deviations of both A and B variables were multiple times of initial SD(A). For example, T2 means both SD of A and B were 2 in (**b**) subplot. In subplot (**c**), the SD of B was multiple times that of the initial SD (A). In subplot (**d**), varied sample size from 500 to 10,000 with an SD of A and B were 1. For example, 1000 means 1000 samples.

Quantifying the potential spurious correlations among the elements of *m*RUE will increase the effectiveness of our *m*RUE model. In this study, we calculated the range of spurious determination coefficients of a manipulative experiment (Fig. 6a). We hypothesized some situations for the dataset that *m*RUE model will encounter in future modelling (Fig. 6b–d). The standard deviation (SD) of common (A) and independent variables (B) were both needed for calculating ε and *m*RUE in all analyses of our Monte Carlo test. The spurious correlations about our manipulative experiment had embedded in the above description (Fig. 6a). As for the possible situations of dataset in the future modelling, we found that there were no significant influences among the different SDs of both A and B (SD = 1, 2, 5) on the relationships between the common variable (A) and ε (Fig. 6b left panel). There was a significant effect between SD of 5 and the other SDs (1, 2) on the relationships between the common variable (A) and *m*RUE (Fig. 6b right panel). When the SD of B was two, five or ten times that of the SD of A, there were significant differences in the range of the spurious determination coefficients among T2, T5 and T10 situations (Fig. 6c). When holding the SD of A and B as constant with random sample sizes of 500–10,000, we found that the sample size produced identical means while yielding a lower variability of spurious determination coefficients along the sample size gradient (Fig. 6d). Thus, the bivariate correlations involving spurious correlations should not be arbitrarily considered meaningless, but should be dealt with case by case.

## Take-Home Message

This study was designed to refine a fundamental concept regarding production in ecosystem studies. Following a newly proposed multiple resource use efficiency model (*m*RUE), we used data from a manipulative experiment to assess the performance of key variables by addressing several key issues. With the experimental data, we found that the water-driven changes on water, light, and nitrogen resources had profound impacts on ecosystem production. The increased available water suppressed ecosystem *mR*UE (i.e., “declining marginal returns”). The variations of *m*RUE caused significant negative effects on ɛ (i.e., “inverse feedback”). This *m*RUE model will facilitate the explanation of the resource uses and ecosystem production in semi-arid grasslands, providing us with promising evidences for broader applications of the *m*RUE in modelling ecosystem production.

## The test-bed and data

The experiment was located in the Duolun Restoration Ecology Station of Inner Mongolia (42°02′N, 116°17′E, 1324 m a.s.l.). This region has a continental monsoon climate. The annual mean temperature is 2.2 °C. The average annual precipitation was about 380 mm, with 67% of annual precipitation occurring in the growing season (May–October). There are enough light, but lacked water and nutrient in soil.

The water-availability gradient experiment was conducted in 2010. This experiment was a randomized block design with seven levels of precipitation treatments: −60% (P−6), −40% (P−4), −20% (P−2), ambient precipitation as a control (CK), +20% (P + 2), +40% (P + 4), and +60% (P + 6), with six replicates for each treatment. The treatments were applied to a total of 42 plots (seven levels and six replications), with 4 × 4 m^2^ for each plot. The intercepted rainfall by rainout shelters was collected to water to the increased-precipitation plots. More details about the measurements were referred to the “Supplementary Information” section.

The relative importance analyses (function “calc.relimp” in “relaimpo” package in R) were performed to test the relative importance values on *m*RUE, ε and ANPP. Prior to our SEM analysis, all variables were calculated for their growing season accumulations (i.e., annual accumulations, see “Supplementary Information”). Non-recursive SEM was constructed to test how the water-driven variables of Ms and Tr affected light and nitrogen, and synthetically affected the multiple resource ratio and efficiency. Prior to the modeling, all variables were log-transformed to assure the homogeneity of variance. In addition, univariate and multivariate normality analysis were performed to meet the model assumptions before SEM analysis using R functions (“skewness” and “kurtosis” in the “moments” package, and “mardia” in the “psych” package, Supplementary Table S1). The maximum likelihood procedure was used to estimate the regression coefficients, variances, covariance and correlations in our SEM. Final model selections were made by the comprehensive results of λ^2^ (P > 0.05), GFI (>0.9), AGFI (>0.9) and RMSEA (<0.05). The package of AMOS 17.0 in SPSS was used for our SEM analysis.

Our *m*RUE model was developed to evaluate ecosystem productivity by involving multiple resources (e.g., light, potassium and phosphor) in an effort of exploring the complex interactions among elements. However, some bivariate analyses would have spurious correlations due to the shared elements (e.g., aPAR and ε), which would magnify the true relationships among them. Consequently, one needs to quantify the contributions of spurious correlations and separate them from the total^41^. In our study, Monte Carlo simulations were performed to quantify the spurious correlations. All the simulations were conducted 1,000 times. In our SEM model, we generated the normal random numbers by using a random number generator (function “rnorm” in R) with a sample size of 126 for mean ± SD of aPAR (10 ± 0.3), PAR (11 ± 0.04), Tr (13 ± 0.3), Ms (15 ± 0.2), N_uptake_ (1.2 ± 0.05), N_avail_ (3.7 ± 0.17) and ANPP (5.3 ± 0.4) in our experiment. Pearson correlations were performed to calculate the range of the spurious determination coefficient (spurious R^2^). In addition, the relative contributions of each common variable on ε or *m*RUE were calculated by the relative importance analyses (function “calc.relimp” in “relaimpo” package in R) to calculate the total spurious contributions of all common variables on ε or *m*RUE. We also performed stochastic simulations to assess the sensitivity of *m*RUE to spurious correlation by changing: (i) both SD of the common variable (A) and the independent variables (B); (ii) the SD of (B) as the multiple times of SD (A); and (iii) sample sizes of 500, 1000, 2000 and 10000 with a constant SD on A and B.

## Additional Information

**How to cite this article**: Han, J. *et al.* Multiple Resource Use Efficiency (*m*RUE): A New Concept for Ecosystem Production. *Sci. Rep.* **6**, 37453; doi: 10.1038/srep37453 (2016).

**Publisher’s note:** Springer Nature remains neutral with regard to jurisdictional claims in published maps and institutional affiliations.

## Supplementary Material

Supplementary Information

## Figures and Tables

**Table 1 t1:** Major acronyms used in this study.

Full name	Abbreviation
Aboveground net primary production	ANPP
Absorbed resource	R
Accumulated absorbed PAR	aPAR
Accumulated soil nitrogen content	Ns
Accumulated soil water content	Ms
Accumulated transpiration	Tr
Accumulated uptake nitrogen from soil	N_uptake_
Available resource	R_avail_
Correlation coefficient	r
Gross primary production	GPP
Light use efficiency	LUE
Multiple resource absorption rate	ε
Multiple resource use efficiency	*m*RUE
Net primary production	NPP
Nitrogen use efficiency	NUE
Photosynthetically active radiation	PAR
Standard coefficient	β
Water availability	Water Avail.
Water use efficiency	WUE

**Table 2:** Summary of publications on resource use efficiency in different ecosystems.

Ecosystem type	Production (g m^−2^ year^−1^)	*m*RUE	Efficiency value	Formula	Location	Publication
Grassland	38–267	WUE	0.035–0.071%	ANPP/PPT	Inner Mongolia	Bai *et al.*^14^
Grassland	(344.14 g C m^−2^ year^−1^)	WUE	(1.15 g C kg^−1^ H_2_O)^a^	GEP/ET	Inner Mongolia	Hu *et al.*^33^
Grassland	100–500	WUE	0.01–0.1%	ANPP/PPT	Central Great Plains	Lauenroth *et al.*^15^
Driest years in arid ecosystems	<500	WUE	convergence to 0.042%	ANPP/PPT	Terrestrial	Huxman *et al.*^34^
Forest	950–3910	WUE	0.134–0.458%	ANPP/PPT	Bahia, Brazil	Stape^32^
Forest	200–2500	LUE	1.4–2.5%	ANPP/aPAR	Savannah River	Allen *et al.*^29^
Cropland		LUE	2.40%	ANPP/aPAR	Britain	Monteith^3^
Grassland	58.9–288.4	LUE	(0.51–0.95 gC MJ^−1^)^b^	NPP/aPAR	Kazakhstan	Propastin *et al.*^35^
Terrestrial	(30–1000 g C m^−2^ year^−1^)	LUE	(0.604–1.259 gC MJ^−1^)^c^	NPP/aPAR	Terrestrial	Running *et al.*^42^
Forest	(122–3125 gC m^−2^ year^−1^)	LUE	(0.4–1.5 gC MJ^−1^)^d^	GEP/aPAR	Terrestrial	Garbulsky *et al.*^5^
Cropland	440–860	NUE	13.1–17.5	ANPP/N_uptake_	Luzon, Philippines	Cassman *et al.*^43^
Forest	757–1393	NUE	307–341	ANPP/N_uptake_	Hawaii, USA	Harrington *et al.*^31^
Grassland	97–121	NUE	177–193	NPP/N_uptake_	Duolun county, Inner Mongolia	Yuan *et al.*^37^
Forest	(2.7–10.8 g m^−2^ d^−1^)	NUE	100–800	NPP/N_uptake_	La Selva, Spain	Hiremath and Ewel^44^
Grassland	221.04 ± 19.04	WUE	0.05% ± 0.0044%	ANPP/Tr	Inner Mongolia	Control plots of this study
		LUE	1.20% ± 0.14%	ANPP/aPAR		
		NUE	62.47 ± 3.08	ANPP/N_uptake_		

We consider the caloric value of terrestrial as ~17.5 KJ g^−1^, thus, LUE: 1 g kJ^−1^ × 17.5 kJ g^−1^ = ^1^7.5 g g^−1^. ^a^~0.25%; ^b^1 gC MJ^−1^ is equal to 0.0175 gC g^−1^, and the carbon element accounts for ~45% of dry matter product, thus, there are ~3.87% (=0.0175/45%) dry matter per light absorbed, thus 0.51–0.95 gC MJ^−1^ was ~1.972–3.673%; ^c^~2.33–4.87%; ^d^ ~1; In the control plot of this study: Mean ± SD of WUE, NUE, LUE were described in our experiment.

**Table 3:** Standard coefficients (β) in decomposed effects based on a non-recursive structural equation (SEM) model.

Independent variable	Endogenous variable							
	aPAR	*P* value	N_uptake_	*P* value	ε	*P* value	*m*RUE	*P* value
tot.Ms								
direct effect	0.58	<0.001			−0.15	<0.001	−0.49	<0.001
indirect effect					0.20	<0.001		
tot.Tr								
direct effect			0.32	<0.001	0.43	<0.001		
indirect effect	0.11	0.002			0.25	<0.001	0.21	
aPAR								
direct effect					0.3	<0.001		
indirect effect								
N_uptake_								
direct effect	0.35	<0.001			0.67	<0.001	0.66	<0.001
indirect effect					0.03	<0.001		
ε								
direct effect							0.23	0.387
indirect effect								
*m*RUE								
direct effect					−0.11	0.014		
indirect effect								

The direct effects of aPAR and Nuptake on ε and *m*RUE were also driven by Ms and Tr.
