# Supplementary material for: Multiple Resource Use Efficiency (mRUE): A New Concept for Ecosystem Production
Source: Sci Rep. 2016 Nov 21;6:37453. doi: 10.1038/srep37453 (PMC5116645; doi:10.1038/srep37453)
Supplement: Supplementary Information [file srep37453-s1.pdf]

Multiple Resource Use Efficiency (*m*RUE): A New Concept for Ecosystem Production

Juanjuan Han[1], Jiquan Chen[2, 1], Yuan Miao[3], Shiqiang Wan[3*]

[1.] International Center for Ecology, Meteorology, and Environment, Nanjing University of Information Science and Technology, Nanjing 210044, China

[2.] CGCEO/Geography, Michigan State University, East Lansing, Michigan 48824, USA

[3.] International Joint Research Laboratory for Global Change Ecology, State Key Laboratory of Cotton Biology, College of Life Sciences, Henan University, Kaifeng, Henan 475004, China

Corresponding author:

Shiqiang Wan

GlobalChangeLab@163.com

Tel: +86 371 23881006

Supplementary Information

1. Non-recursive structure equation model

To focus on the critical ecosystem processes and simplify the model, some cues were not included, leading to the degree of uncertainty on some dependent variables. For example, only 54% of *m*RUE (multiple-resource use efficiency model) was explained, which implied that other factors should be involved in to contribute, such as community richness, land use history and legacies of extreme events.

2. Multiple-resource use efficiency model

To keep consistent with the unit of ANPP, all the variables relevant to R (aPAR, Tr, N_{uptake}) and R_{avail} (PAR, Ms, Ns) should be converted to their accumulative forms (unit: g m^{-2} $year^{-1}$) at annual scales. Here, the values of Ms and Ns need to accumulate to the depths of 0–30 cm and the area of 1 m^2. N_{uptake} was calculated by the plant nitrogen content multiplied by the aboveground biomass per 1 m^2 quadrat. For accumulated Tr, in our experiment, ecosystem evapotranspiration and soil evaporation were measured by an Li-6400 three times a month. Plant transpiration was the difference of evapotranspiration and evaporation. Accumulated transpiration was calculated by the product of each measured transpiration and the interval time between the two measuring dates on 1 m^2 soil areas. PAR and aPAR should convert their units and then divide by community caloric values[1] (19.38 kJ g^{-1}). The aPAR is a function of community cover. We first calculated PAR by subtracting community leaf reflectance and transmittance from 1 and then multiplying by the community cover and the zenith angle of $\cos 30^{o}$. The zenith angle 30° was a good representation for the mean intercepted radiation of the whole day[2]. We set the sum value of leaf reflectance

and transmittance as 0.29 [4].

NUE can be defined as the inverse of plant nitrogen concentration in grassland[3]. LUE (light use efficiency) and WUE (water use efficiency) were calculated by dividing ANPP by aPAR or Tr, respectively.

3. The details in experimental measurements

ANPP was sampled by harvesting the aboveground living biomass of the two 0.15 cm^2 quadrats in each plot during the middle of August every year (2010-2012). Plant cover was measured by species in mid-August every year. The samples of soil and plants were analyzed by an Vario EL III to obtain the total nitrogen content. Soil moisture was simultaneously measured by evaporation and evapotranspiration with a portable soil moisture device (Diviner 2000, Sentek Pty Ltd, Balmain, Australia). Incidence PAR was real-time monitored by Li190SB (Campbell Scientific, Logan, UT, USA), rainfall was monitored by TE525MM (Campbell Scientific, Logan, UT, USA). These two sensors were installed on an eddy covariance tower near our experiment. Ecosystem fluxes ($0.5\times0.5\times0.5\ m^3$), such as ecosystem evapotranspiration and soil evaporation, were measured almost every ten days during 2010–2012 by an Li-6400 (IRGA; LI-6400, Li-Cor, Lincoln, NE, USA).

Reference

1 Chen, Z. & Zhang, H. The caloric values of the steppe plants in the typical steppe zone of Inner Mongolia. *Chinese Bulletin of Botany*, 26-27 (1993).

2 Allen, C. B., Will, R. E., McGarvey, R. C., Coyle, D. R. & Coleman, M. D.

Radiation-use efficiency and gas exchange responses to water and nutrient availability in irrigated and fertilized stands of sweetgum and sycamore. *Tree Physiol.* **25**, 191-200 (2005).

3 Chapin III, F. S. The mineral nutrition of wild plants. *Annu Rev Ecol Syst*, 233-260 (1980).

4. Radiation transfer model intercomparison (RAMI), Jobs, Growth and Investment-European Commission; available at http://rami-benchmark.jrc.ec.europa.eu/HTML/RAMI4PILPS/OVERVIEW/GRASSLANDS/GRASSLANDS.php

Table S1. Assessment of normality in the SEM model. To meet multivariate assumptions of the SEM model, absolute values of skew and kurtosis should be less than 3 and 8, respectively. P-value of multivariate kurtosis (0.062) was insignificant from zero.

Variable	mean	min	max	skew	kurtosis	*P*-value
Ms	5319256	2367650	7797280	-0.671	2.792	0.79
Tr	455446	165097	789393	-1.143	3.936	0.051
aPAR	17908	7676	29337	-0.379	2.647	0.467
N_{uptake}	3.525	0.626	7.244	-1.251	5.339	0.001
ε	0.114	0.055	0.157	-0.989	3.815	0.074
*m*RUE	0.071	0.03	0.119	-0.497	4.094	0.031
Multivariate					1.86	0.062
